# Profound hyponatremia in cirrhosis: a case report

**DOI:** 10.1186/1757-1626-3-77

**Published:** 2010-03-23

**Authors:** Aaron Lindsay

**Affiliations:** 1 Case Western Reserve University, School of Medicine, 10900 Euclid Ave, Cleveland, OH 44106, USA

## Abstract

**Introduction:**

Cirrhosis of the liver commonly leads to a state of chronic hypervolemic hyponatremia. Profound exacerbation of the hyponatremic state may occur in patients with decompensated cirrhosis in conjunction with acute stressors such as infection or binge alcohol ingestion.

**Case presentation:**

A 47 year old man with a history of alcoholic cirrhosis presented to the hospital with symptomatic profound hyponatremia (serum sodium concentration of 105 meq/L) due to a recent infection and binge drinking. The patient was treated with antibiotics, diuretics and hypertonic saline and was placed on a fluid restricted diet. The serum sodium level corrected slowly over four days with symptomatic improvement occurring after two days. A brief discussion of the symptoms and treatment of acute and chronic hyponatremia in the setting of cirrhosis is included.

**Conclusion:**

In patients with cirrhosis, it is important to recognize the symptoms of hyponatremia, identify and treat any exacerbating conditions early in their course, and correct the serum sodium concentration slowly with frequent monitoring.

## Introduction

Cirrhosis of the liver frequently leads to a state of chronic hypervolemic hyponatremia. The cause of the hyponatremia is related to a decrease in systemic vascular resistance [1], which is more prominent in the splanchnic circulation, and compensatory neurohormonal mechanisms that are activated due to the hemodynamic changes. The hyponatremia may be worsened in the setting of binge alcohol drinking which leads to the ingestion of large amounts of fluid containing little sodium [2]. Hyponatremia in cirrhosis can be a severe problem and has been shown to be an independent predictor of mortality in patients waiting for liver transplantation [3,4]. Here is presented a case of a patient with alcoholic cirrhosis who presents to the hospital with a serum sodium concentration of 105 meq/L.

## Case presentation

A 47-year-old Caucasian male was admitted with a three day history of shortness of breath, weakness, anorexia, and unsteady gait, including a recent fall. The patient had a medical history significant for peptic ulcer disease, hypertension, and alcoholic cirrhosis. His family history was not significant. He reported smoking 2 packs per day and drinking 8-12 beers per day for the past 20 years. Over the previous 6 months, the patient had been unemployed and reported increased alcohol consumption. On physical exam, the patient was noted to have marked ascites with a prominent fluid wave and bulging flanks, bilateral pitting edema above the knees, and crackles as well as dullness to percussion in the left lower lobes. In addition, chest radiographs revealed a left lower lobe pneumonia. Significant laboratory studies included a WBC of 19.4 × 10^9^/L, AST of 107 U/L, ALT of 66 U/L, and serum sodium concentration of 105 meq/L. The patient was treated with IV furosemide (40 mg × 1 dose) and 3% NaCl in addition to fluid restriction because of his symptomatic and profound hyponatremia. His serum sodium concentration increased to 120 meq/L over 48 hours and 130 meq/L over 96 hours with the use of 3% NaCl and fluid restriction, and his weakness, anorexia, and unsteady gate improved. His pneumonia was treated with Vancomycin (1 g IV every 12 hrs) and Piperacillin/Tazobactam (3.375 mg IV every 6 hrs) which resulted in symptomatic and radiographic improvement. He was discharged from the hospital on oral spironolactone (200 mg/day) and furosemide (40 mg/day) with recommendations for abstinence from alcohol, a low sodium diet, and fluid restriction.

## Discussion

Chronic hyponatremia (defined as a serum sodium concentration below 130 meq/L) occurs in up to 22 percent of people with cirrhosis [5], and is often asymptomatic if the serum sodium concentration is above 120 meq/L [6,7]. The percent of people with cirrhosis affected by chronic hyponatremia increases to 50 percent if a cutoff for serum sodium concentration of 135 meq/L, the lower limit of normal, is used [5]. The frequency of such profound hyponatremia as seen at presentation in the patient in this case (serum sodium concentration of 105 meq/L) is unclear; a population survey reported that the prevalence of patients with cirrhosis and serum sodium concentrations less than or equal to 120 meq/L is 1.2 percent [6].

The most common reason for chronic hyponatremia in cirrhosis is impairment in renal solute-free water secretion due to increased antidiuretic hormone secretion and decreased effective arterial volume [5,8]. The brain is able to compensate for the increased osmolar pressure (which leads to cerebral edema) in chronic hyponatremia by extruding intracellular osmolytes, such as potassium, glutamine and myoinositol, which can take 48 hours for full effect [5,7,9]. This adaptive mechanism explains why patients with chronic hyponatremia and serum sodium concentrations above 120 meq/L are often asymptomatic.

If there is an acute exacerbating condition however, such as infection or binge drinking as in this patient, the serum sodium concentration can rapidly drop to dangerously low levels for which the brain is unable to compensate acutely. Acute worsening of chronic hyponatremia may have various etiologic factors, including: generalized haemodynamic derangement with low peripheral resistance, reduced effective arterial volume, hypothalamic overproduction of antidiuretic hormone, elevated renin and angiotensin, hypotonic fluid ingestion and reduced glomerular filtration, all of which may lead to marked water retention [5,7,8]. The ingestion of large amounts of alcohol, a hypotonic fluid containing little sodium, may worsen the underlying chronic hyponatremia present in cirrhosis [2] and is believed to be a factor for the acute decompensation seen in this patient.

Common symptoms of chronic hyponatremia include fatigue, nausea, dizziness, lethargy, confusion, and gait disturbances [10,11]. In the setting of acute hyponatremia, or acute on chronic hyponatremia, seizures, coma, and respiratory arrest may occur [10]. It should be noted that the symptoms experienced by the patient presented in this report are likely multifactorial and due to both the profound hyponatremia and comorbid lobar pneumonia. Treatment of hyponatremia in patients with cirrhosis includes sodium and fluid restriction and continued treatments with spironolactone and loop diuretics. However, care must be taken when administering diuretics as they can exacerbate the reduction in tissue perfusion that occurs in cirrhosis, further impairing the ability to excrete free water [12]. Bolus infusions of 3% NaCl are reserved for patients with profound hyponatremia and severe symptoms [13]. Vasopressin receptor antagonists are newer therapies and their precise role in treating hypervolemic hyponatremia in patients with cirrhosis is not well-defined [5,7]. The serum sodium concentration in symptomatic hyponatremia should be corrected at a rate no more than 10 meq/L in the first 24 hours, 18 meq/L in the first 48 hours, and 20 meq/L in the first 72 hours to prevent iatrogenic brain injury and central pontine myelinolysis [13,14]. In patients with cirrhosis, it is important to recognize the symptoms of hyponatremia, identify and treat any exacerbating conditions early in their course, and correct the serum sodium concentration slowly with frequent monitoring.

## Abbreviations

ALT: alanine transaminase; AST: aspartate transaminase; IV: intravenous; NaCl: Sodium Chloride; WBC: white blood cell count.

## Consent

Written informed consent was obtained from the patient for publication of this case report. A copy of the written consent is available for review by the Editor-in-Chief of this journal.

## Competing interests

The author declares that he has no competing interests.
